# Antibiotic treatment in feedlot cattle: a longitudinal study of the effect of oxytetracycline and tulathromycin on the fecal and nasopharyngeal microbiota

**DOI:** 10.1186/s40168-019-0696-4

**Published:** 2019-06-05

**Authors:** Devin B. Holman, Wenzhu Yang, Trevor W. Alexander

**Affiliations:** 1 Lacombe Research and Development Centre, Agriculture and Agri-Food Canada, Lacombe, AB Canada; 2 Lethbridge Research and Development Centre, Agriculture and Agri-Food Canada, Lethbridge, AB Canada

**Keywords:** Nasopharyngeal microbiome, Fecal microbiome, Antibiotic resistance, Oxytetracycline, Tulathromycin, Feedlot cattle

## Abstract

**Background:**

Beef cattle in North America frequently receive an antibiotic injection after feedlot placement to control and manage bovine respiratory disease. The potential collateral effect of these antibiotics on the bovine microbiome is largely unknown. Therefore, we determined the longitudinal impact of two commonly administered veterinary antibiotics, oxytetracycline and tulathromycin, on the fecal and nasopharyngeal (NP) microbiota of beef cattle that were transported to a feedlot. We also report the effect these antibiotics have on several antibiotic resistance determinants in both the fecal and NP microbiome.

**Results:**

Oxytetracycline and tulathromycin perturbation of the bovine fecal and NP microbiota was greatest at days 2 and 5. Although the NP microbiota of the tulathromycin-treated cattle had recovered by day 12, the NP microbiota of the oxytetracycline-treated group remained altered through day 34. Overall, the NP microbiota appeared to be more sensitive to antibiotic treatment than the fecal microbiota. Members of the bacterial *Microbacteriaceae* family were most notably affected by antibiotic administration in the NP microbiota. Both antibiotics protected against *Pasteurella* spp. in the nasopharynx at days 2 and 5. Despite very similar diets at both locations, the largest shift in the fecal and NP microbiota occurred after transport to the feedlot (*P* < 0.05). Antibiotic resistance determinants in the NP microbiome were also affected more strongly by antibiotic treatment than those in the fecal microbiome. Oxytetracycline increased the proportion of *erm*(X), *sul2*, *tet*(H), *tet*(M), and *tet*(W) in NP samples and *tet*(M) and *tet*(W) in fecal samples, at day 12 (*P* < 0.05). The effect of tulathromycin on the relative abundance of resistance genes in the NP microbiome was greatest at day 34 as *erm*(X), *sul2*, and *tet*(M) were enriched (*P* < 0.05).

**Conclusions:**

Administration of a single injection of oxytetracycline and tulathromycin resulted in significant changes in the NP and fecal microbiota during the first 5 days after treatment. Antibiotic treatment also increased the relative abundance of several antibiotic resistance determinants in the fecal and NP microbiome at either day 12 or 34.

**Electronic supplementary material:**

The online version of this article (10.1186/s40168-019-0696-4) contains supplementary material, which is available to authorized users.

## Background

Bovine respiratory disease (BRD), also called shipping fever, remains the most common cause of morbidity and mortality after feedlot placement [1], resulting in significant economic losses [2]. It is a multifactorial disease but bacterial species, including *Bibersteinia trehalosi*, *Histophilus somni*, *Mannheimia haemolytica*, *Mycoplasma bovis*, and *Pasteurella multocida*, are frequently implicated [3]. The upper respiratory tract is a reservoir of these opportunistic pathogens, which can proliferate and infect the lungs when cattle immunity is compromised due to stress or primary viral infections [4]. High-risk cattle populations (recently weaned, lightweight, commingled, auction market derived, etc.) entering feedlots are most susceptible to BRD. As a result, cattle are often administered metaphylactic antibiotics via subcutaneous injection to treat existing lung infections at the time of entry and prevent infections after feedlot placement. In the USA for example, the macrolide tulathromycin was reported to be used as metaphylaxis in 45.3% feedlots at placement and oxytetracycline in 17.4% (USDA, 2013).

Pathogenic bacteria that can be cultured in the laboratory have been the main focus of research on the bovine respiratory tract until very recently. However, there is increasing awareness regarding the importance of the mammalian microbiome in relation to health and it is clear that the resident microbiota of the respiratory tract have a critical role in preventing colonization of pathogens [5, 6]. The establishment and stability of the mammalian respiratory microbiota is critical to health and disruption can predispose to infection [7]. Transportation to a feedlot [8] and diet composition [9] have previously been shown to affect the nasal microbiota of beef calves, highlighting that respiratory bacteria of cattle are perturbed by industry management practices. Metaphylactic antibiotic administration may also potentially affect the bovine respiratory and gut microbiota. In humans, antibiotic use has been linked to an altered microbial community structure in the upper respiratory tract of children for up to 6 months after administration [10], showing that a prolonged antibiotic effect takes place. Recently, we observed specific changes in the nasopharyngeal (NP) microbiota of commercial beef cattle that received an injectable antibiotic at feedlot entry; however, cattle were not sampled longitudinally to evaluate the microbiota while the antibiotics were bioactive [11].

Antibiotic-driven alterations in the respiratory microbiota of cattle could have implications for the management of cattle in feedlots if metaphylactic antibiotic use provides a favorable niche for select pathogens. Indeed, bacteria from the bovine upper respiratory tract have been identified that can act to either inhibit or enhance growth of *M*. *haemolytica*, *P*. *multocida*, and *H*. *somni* [12]. In addition, we have previously noted an increase in *Mycoplasma* spp. after feedlot placement, although factors leading to this proliferation are unknown [8]. Presumably, injectable antibiotics also affect non-respiratory bacteria, depending on the pharmacokinetics of the drug. For example, when injected, approximately half of tulathromycin is eliminated unaltered by biliary excretion [13] and up to 27% of oxytetracycline is excreted non-renally [14] and therefore microbes in the lower gastrointestinal tract of cattle are exposed to these antibiotics as well.

No research to date has examined the effect of injectable antibiotics on the NP microbiota of beef cattle in a controlled setting. Furthermore, the impact that these antibiotics have on the gut microbiota of feedlot cattle is also largely unknown. Development of resistance in human and animal pathogens resulting from antibiotic use is a serious issue in human and veterinary medicine. The level of resistance in a microbial community also affects the microbial community dynamics following antibiotic challenge, with resistant bacteria potentially outcompeting susceptible bacteria. Depending on distribution, metabolism, and excretion of the antibiotic, bacterial communities throughout the host could be affected. There is also a concern that animals treated with antibiotics may shed resistant bacteria and resistance determinants into the surrounding environment [15].

Therefore, in the present study, we used cattle from a closed herd to investigate the effect of injectable antibiotics on the NP and gut microbiota of beef cattle after feedlot placement. Based on our previous work demonstrating the relative instability of the NP microbiota of feedlot cattle [8], we hypothesized that injectable antibiotics would have a larger effect on the NP microbial community than on the fecal microbiota. We also wanted to know if the NP and/or fecal microbiota would recover following a single antibiotic injection and if resistance determinants would be enriched in the microbiome of these treated animals.

## Methods

Animals in this experiment were cared for in agreement with the Canadian Council for Animal Care (2009) guidelines. The Animal Care Committee at the Lethbridge Research and Development Centre reviewed and approved all procedures and protocols.

### Animals and experimental design

The cattle in this study were sourced from a previously described closed research herd that had a complete history of health and antibiotic use available [8]. Thirty-six Angus × Herford steers (*n* = 18) and heifers (*n* = 18) were selected for inclusion in the study based on weight (300.8 ± 3.6 kg). Cattle were transported (d minus 2) from the research farm to the Lethbridge Research and Development Centre feedlot, a distance of 20 km. Upon arrival at the feedlot, the cattle were blocked by body weight and sex and then randomly assigned to one of three treatment groups (*n* = 12), control, oxytetracycline, and tulathromycin. The animals were allowed to acclimate to the feedlot environment for 2 days prior to administration of antibiotics and the first feedlot sampling. Cattle were not exposed to antibiotics or vaccinated prior to the study and they did not receive hormone implants. The animals were weighed just prior to transport and at day 34.

**Fig. 1:**
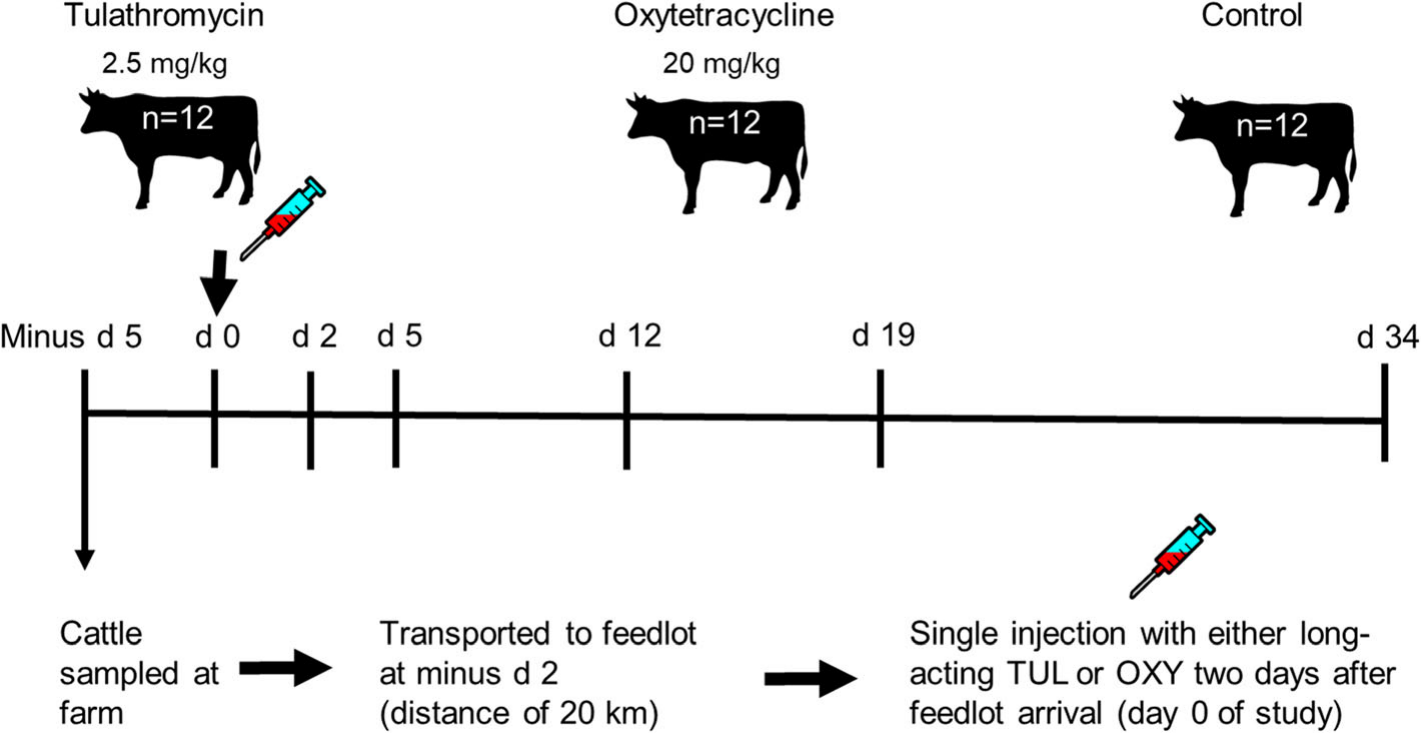
Timeline for fecal and nasopharyngeal sampling. Experimental sampling days are indicated above the line and antibiotic injection is noted at day 0. The number of animals in each treatment group is displayed at the top of the figure

On day 0, fresh fecal and nasopharyngeal swabs [8] were obtained while animals were restrained in a squeeze chute. Following sampling, cattle in the antibiotic treatment groups received either a single injection of long-acting oxytetracycline (20 mg/kg body weight) or tulathromycin (2.5 mg/kg body weight). All cattle were then sampled at days 2, 5, 12, 19, and 34 (Fig. 1). Samples were also taken from the cattle at the research farm 3 days prior to transport (d minus 5). All fecal and nasopharyngeal samples were immediately placed on ice and then stored at − 80 °C until DNA extraction.

Barley silage was fed to the animals at both the research farm and feedlot. The dry matter (DM) content of the silages from the farm and feedlot were determined by drying 50 g of each sample at 55 °C in a forced-air oven for 48 h. Subsamples collected on each sampling day were lyophilized and ground with a 1-mm screen using a Wiley mill for determination of organic matter (OM) according to the procedure of AOAC [16] (method 942.05). Neutral detergent fiber (NDF) was analyzed with the addition of sodium sulfite (10 g/L) and acid detergent fiber analysis (ADF) was analyzed only with ADF solution accordingly to the procedure of AOAC [16], using an Ankom 200 system (Ankom Technology Corporation, Fairport, NY, USA). Total nitrogen content was analyzed by using elemental analysis (NA1500 Nitrogen/Carbon analyzer, Carlo Erba Instruments, Milan, Italy).

### Extraction of DNA from fecal and nasopharyngeal samples

Total microbial DNA was extracted from 200 mg of each fecal sample using the QIAamp Fast DNA stool mini kit (Qiagen Inc., Toronto, ON, Canada) according to manufacturer’s instructions. A bead-beating step using 300 mg of 0.1 mm zircon/silica beads was included following the addition of InhibitEX buffer and samples were agitated in a Tissuelyser II (Qiagen Inc.) for 5 min at 30 Hz. The Qiagen DNeasy Tissue kit (Qiagen Inc.) was used to extract microbial DNA from the nasopharyngeal swabs as previously detailed [17]. Briefly, this extraction method also included a 5-min bead-beating step at 30 Hz with 300 mg of 0.1 mm zircon/silica beads. The concentration of eluted DNA was measured using the Quant-iT PicoGreen dsDNA Assay Kit (Thermo Fisher Scientific, Ottawa, ON, Canada) and a NanoDrop 3300 Fluorospectrometer (Thermo Fisher Scientific). Negative extraction controls were also included in triplicate for both the fecal and nasopharyngeal extraction kits.

### 16S rRNA genes sequencing and analysis

The 16S rRNA gene libraries were generated as described in Holman et al. [8] with the exception that the modified primers 515-F (5′-GTGYCAGCMGCCGCGGTAA-′3) and 806-R (5′-GGACTACNVGGGTWTCTAAT-′3) were used to target the V4 hypervariable region [18]. Amplicons were sequenced on an Illumina MiSeq instrument (Illumina Inc., San Diego, CA, USA) using the MiSeq reagent kit v2 (500 cycles) as per manufacturer’s instructions.

The software package DADA2 v. 1.4 [19] was used in R v. 3.4.2 [20] to process the 16S rRNA gene sequences. The forward and reverse reads were each truncated at a length of 205 bp and the sequences quality-filtered using a maximum expected error of 2 with no ambiguous bases allowed. The naive Bayesian RDP classifier [21] and the SILVA SSU database v. 128 [22] with a 50% bootstrap confidence threshold were used to assign taxonomy to the quality-filtered merged sequences, referred to hereafter as operational taxonomic units (OTUs) at 100% similarity. The inverse Simpson’s diversity index and OTU richness were calculated in QIIME v. 1.9.1 [23] and Bray-Curtis dissimilarities were assessed using the R packages vegan v. 2.4.3 [24] and phyloseq v. 1.20.0 [25]. OTUs that were predominantly found in the fecal or NP negative extraction controls were removed prior to analysis.

### Quantification of antibiotic resistance determinants

Genes conferring resistance to aminoglycosides (*str*), beta-lactams (*bla*_ROB_ and *bla*_TEM_), macrolides [*erm*(A), *erm*(X)], sulfonamides (*sul2*), and tetracyclines [*tet*(C), *tet*(H), *tet*(M), *tet*(W)] were quantified by real-time PCR and then normalized by 16S rRNA gene copy number in each sample. The primer sequences used were as previously published in Looft et al. [26]. Each real-time PCR reaction consisted of 1X iQ SYBR Green Supermix (Bio-Rad Laboratories Ltd., Mississauga, ON, Canada), 0.4 μM of each primer, 0.1 μg/μl BSA (New England Biolabs, Pickering, ON, Canada), and 25 (NP samples) or 10 (fecal samples) ng of DNA, in a total volume of 25 μl. A CFX96 Touch Real-Time PCR Detection system (Bio-Rad Laboratories Ltd) was used to quantify each gene with the following conditions: an initial denaturation at 95 °C for 3 min, followed by 40 cycles at 95 °C for 25 s, 60 °C for 30 s, and then 72 °C for 45 s. Standard curves (10^2^ to 10^6^ gene copies) were produced for each resistance gene using the pDrive cloning vector (Qiagen Inc.) containing the PCR product from each respective gene. A melt curve analysis was performed following amplification for all real-time PCR reactions to ensure only target genes were amplified.

### Statistical analysis

The NP samples were randomly subsampled to 7300 sequences and the fecal samples to 10,000 sequences, prior to the calculation of the diversity metrics and Bray-Curtis dissimilarities. The number of sequences per sample was chosen to keep as many samples as possible in the analysis. In R v. 3.4.2, a linear mixed model using the lmer function in the lme4 v 1.1.12 package [27] was used to compare diversity measures and resistance determinants by time and treatment. The linear mixed model included the random effect of the individual animal and the fixed effects of treatment, sampling time, sex, and their interactions as fixed effects. Post-hoc comparisons were performed within each sampling time using Tukey’s honestly significant difference (Lenth, 2016). The fecal and nasopharyngeal microbial community structure was analyzed with vegan using permutational multivariate analysis of variance (PERMANOVA; adonis function) with 10,000 permutations. Differentially abundant OTUs among the control and antibiotic treatments and between minus d 5 and d 0 were identified using DESeq2 [28]. For the DESeq2 analysis, samples were not randomly subsampled and only OTUs found in at least 25% of samples were included. The Benjamini-Hochberg procedure was used to control the false discovery rate (FDR). The ADF, DM, OM, NDF, and nitrogen content of the silages at the farm and feedlot were compared using an unpaired *t* test.

## Results

### Diet composition, animal weight gain, and 16S rRNA gene sequencing overview

On a percentage basis, the DM (farm, 92.5 ± 0.1; feedlot, 93.0 ± 0.3), OM (farm, 91.9 ± 0.4; feedlot, 93.1 ± 0.1), NDF (farm, 50.6 ± 1.5; feedlot, 47.4 ± 2.4), and nitrogen (farm, 1.8 ± 0.04; feedlot, 2.0 ± 0.02) contents were similar between silages fed to cattle at the farm and at the feedlot (*P* > 0.05). Only ADF content (farm, 29.5 ± 0.6; feedlot, 20.9 ± 1.4) differed between the silages (*P* < 0.05). The cattle were weighed prior to the start of the study and again at the conclusion (day 34). The growth rate of the cattle was not affected by treatment (*P* > 0.05) and the average weight gain was 32.4 kg ± 1.5 SEM during the research period. All cattle remained healthy throughout the study and did not receive any additional antibiotics. The number of sequences per sample prior to random subsampling and after processing ranged from 7387 to 53,419 and averaged 25,976 ± 333 SEM sequences per sample.

### Antibiotic and longitudinal effects on the nasopharyngeal microbiota

**Fig. 2:**
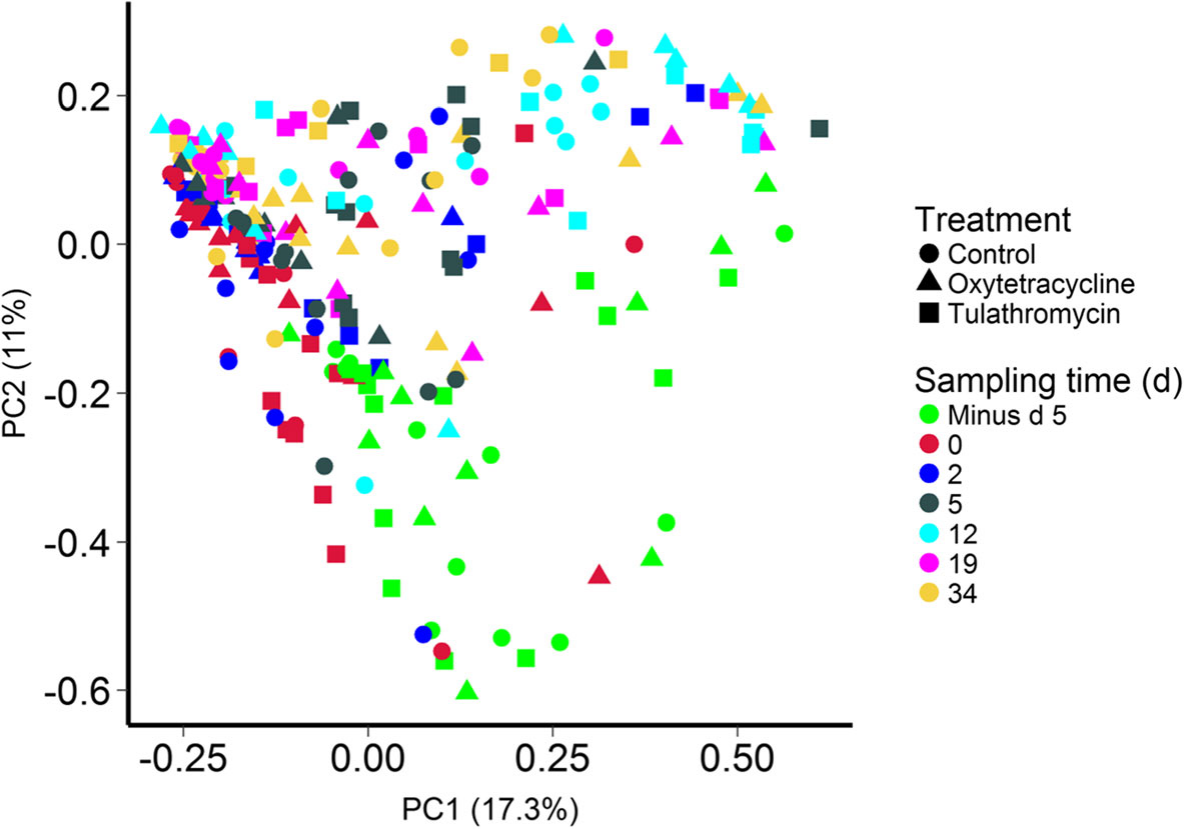
Principal coordinates analysis plots of the Bray-Curtis dissimilarities in nasopharyngeal samples by sampling time (colors) and treatment group (shapes). The percentages of variation explained by the principal coordinates are indicated on the axes

**Fig. 3:**
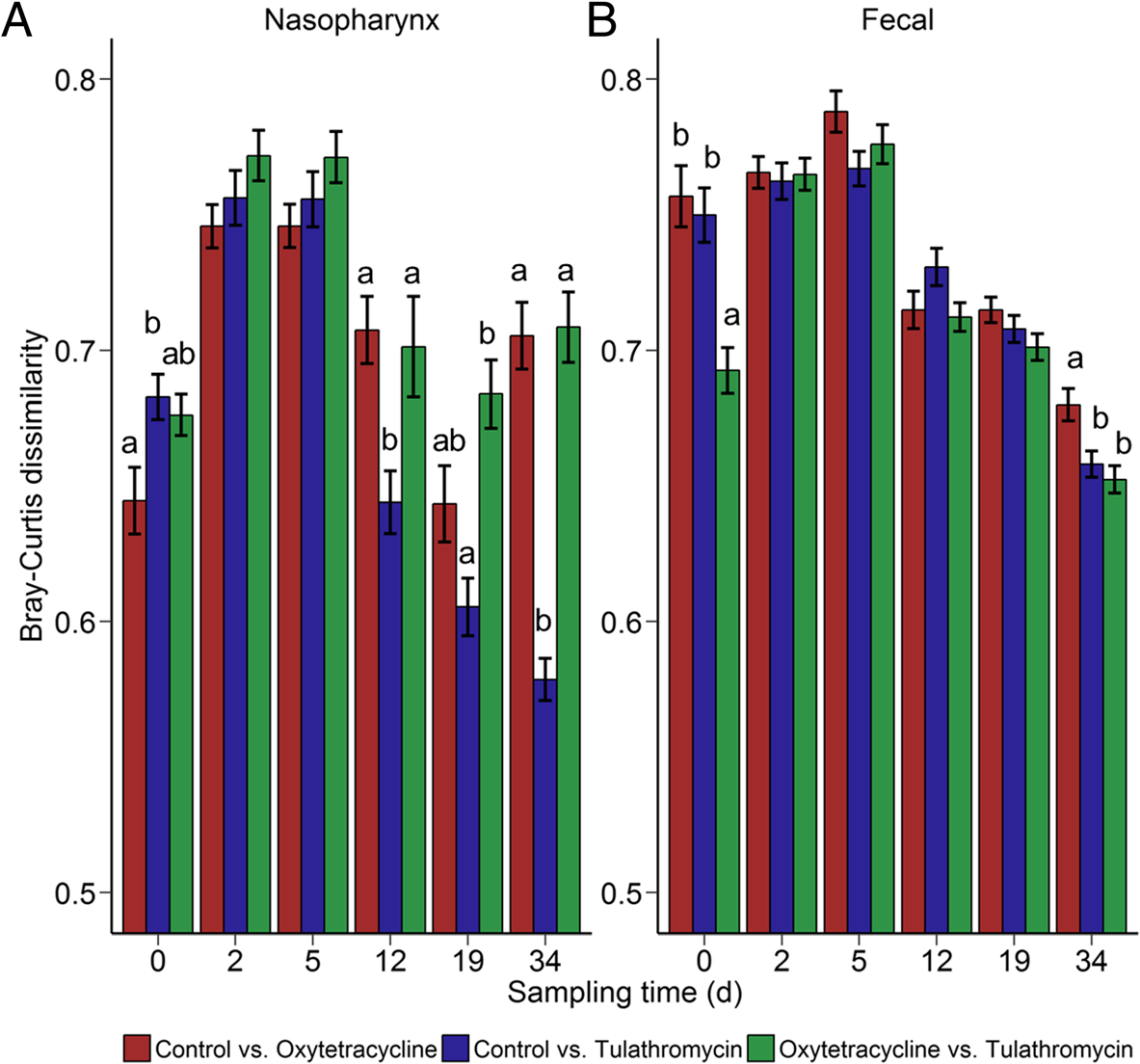
Bray-Curtis dissimilarities between each treatment group at each sampling time for **a** nasopharyngeal and **b** fecal samples. Treatment comparisons with higher values are more dissimilar to each other. Different lowercase letters within each sampling time represent significantly different means (*P* < 0.05). Error bars indicate ± standard error of the mean

The nasopharyngeal microbiota shifted during the initial 5-day period when the cattle were transported from the research herd to the feedlot and prior to antibiotic administration for the tulathromycin and oxytetracycline groups (Fig. 2). This shift was largely driven by a decrease in OTUs classified as members of the *Filobacterium*, *Moraxella*, *Mycoplasma*, and *Pasteurella* genera and an increase in *Acinetobacter*, *Clostridium*, *Corynebacterium*, *Psychrobacter*, *Streptococcus*, and *Ruminococcaceae* UCG-005 OTUs (Additional file 1: Table S1). After animals were placed in the feedlot, there was still a significant time effect on the NP microbiota structure from day 0 to 34 (PERMANOVA: R2 = 0.15; *P* = 0.0001). Treatment with either oxytetracycline or tulathromycin at day 0 also had a significant effect on the NP microbiota (*R*^2^ = 0.044; *P* = 0.00001); however, there was still a larger time effect (*R*^2^ = 0.090; *P* = 0.00001) from day 2 to 34. The NP microbiota of the three treatment groups was most dissimilar at day 2 and 5 (Fig. 3a). From day 12 through 34, the NP microbiota of the tulathromycin-treated animals more closely resembled that of the control group, indicating recovery. However, the NP microbiota of oxytetracycline-treated cattle remained disturbed for the duration of the study in comparison with both the tulathromycin-treated and control animals (*P* < 0.05).

**Fig. 4:**
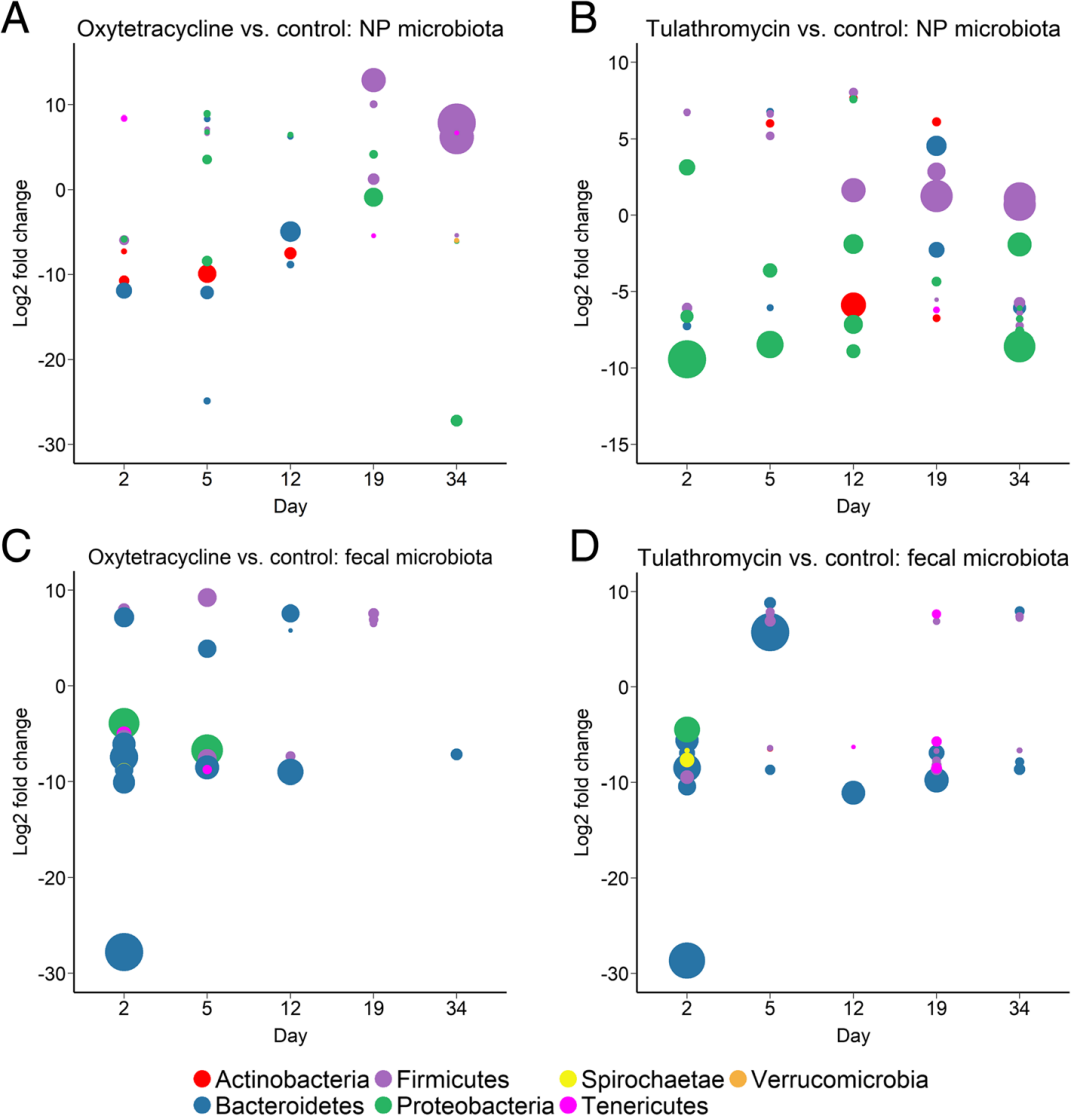
Differentially abundant OTUs between the oxytetracycline (**a**, **c**) and tulathromycin (**b**, **d**) treated and the control cattle for the nasopharyngeal (NP) and fecal microbiota. Each circle is colored by phylum and represents a single OTU with the mean count number for each OTU indicated by the relative size. OTUs with positive log_2_ fold changes were more abundant in the control cattle

We also determined which OTUs were differentially abundant between each antibiotic treatment group and the control cohort throughout the experiment. In the oxytetracycline group, at days 2 and 5, antibiotic treatment was associated with a significant decrease in *Pasteurella* and *Mycoplasma* OTUs at day 2 (FDR < 0.05; Fig. 4; Additional file 1: Table S2). Notably, tulathromycin treatment also reduced the abundance of this same *Pasteurella* OTU at days 2, 5, and 12 (Additional file 1: Table S3). An abundant OTU classified at the family level as *Microbacteriaceae* was enriched in the control group from day 2 through 12 in comparison with the oxytetracycline cohort and at day 12 in relation to the tulathromycin-treated cattle. At day 34, a *Mycoplasma* OTU was actually significantly higher in the NP microbiota of the oxytetracycline-treated cattle. Although two *Moraxella* OTUs were significantly reduced in the tulathromycin-treated animals at day 34, another OTU classified as *Moraxella* (> 10% relative abundance) was enriched in the NP microbiota of the oxytetracycline group at both days 19 and 34 compared with the control cattle (FDR < 0.05; Additional file 1: Table S3). Additionally, the abundance of a Sphingobacteriales OTU was significantly reduced in the oxytetracycline-treated cattle at days 2, 5, and 12.

The OTU richness and the inverse Simpson’s diversity index increased significantly within the NP microbiota of all groups following transport to the feedlot (*P* < 0.0001) (Additional file 2: Figure S1). There were significantly fewer OTUs (*P* < 0.05) at day 19 in the NP microbiota of the oxytetracycline treated cattle compared with the control animals. Interestingly, at days 2 and 5, the inverse Simpson’s diversity was actually higher in the NP microbiota of oxytetracycline cattle in relation to the control and tulathromycin cattle (*P* < 0.05). Although *Moraxella* and *Mycoplasma* were the most relatively abundant genera overall, there was considerable inter-animal variability among the ten most relatively abundant genera (Additional file 3: Figure S2) with the NP microbiota of several animals being dominated (> 50%) by a single genus at a specific sampling time.

### Antibiotic and longitudinal effects on the fecal microbiota

**Fig. 5:**
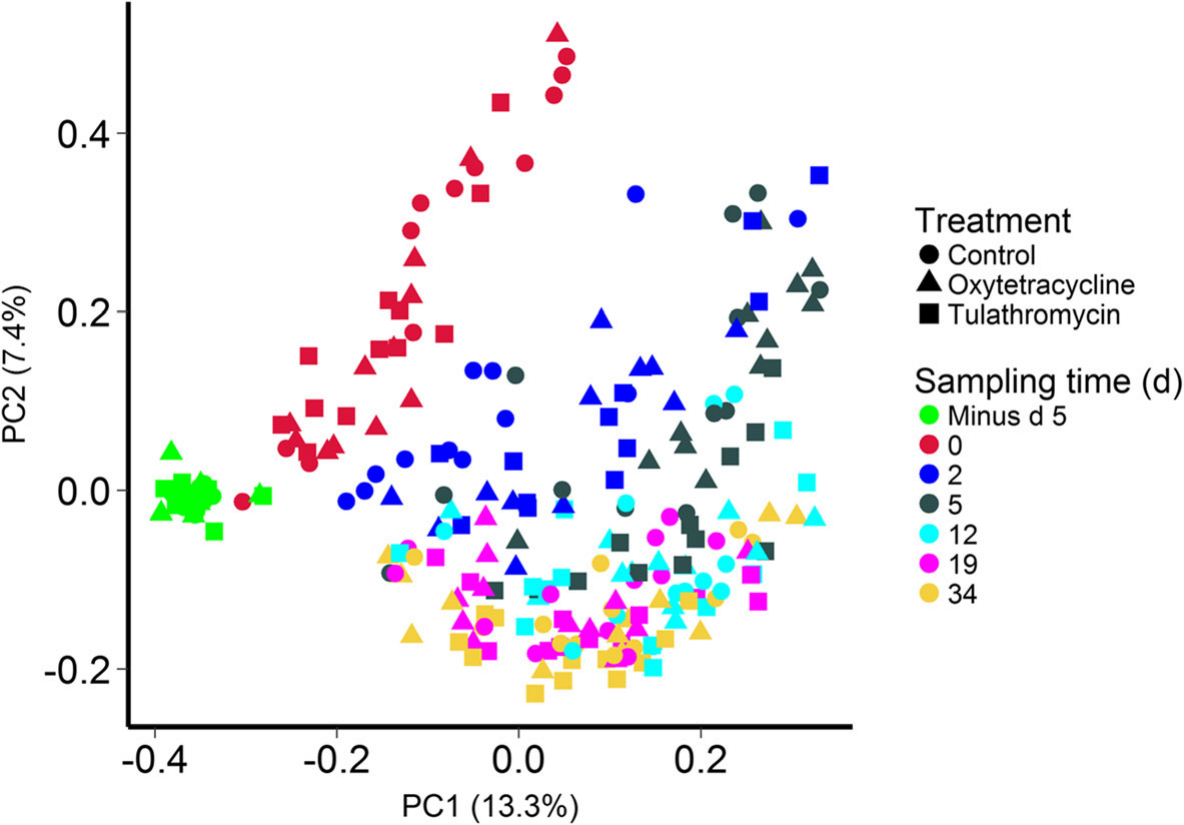
Principal coordinates analysis plot of the Bray-Curtis dissimilarities in fecal samples by sampling time (colors) and treatment group (shapes). The percentages of variation explained by the principal coordinates are indicated on the axes

Transport to the feedlot also resulted in a shift in the microbial community structure of the fecal microbiota (Fig. 5). The fecal microbiota among cattle prior to transport were remarkably similar to each other in comparison with samples taken just 5 days later. A significant increase in the abundance of OTUs classified as *Clostridium*, *Escherichia/Shigella*, *Prevotella*, *Prevotellaceae* YAB2003 group, *Rikenellaceae* RC9 gut group, and *Treponema* contributed to this large shift from d minus 5 to day 0, as did a decrease in *Alistipes*, *Desulfovibrio*, *Phocaeicola*, and *Ruminococcaceae* OTUs (FDR < 0.05; Additional file 1: Table S4). Based on the PCoA plot of the Bray-Curtis dissimilarities, the fecal microbiota continued to visibly change until at least day 12 (*R*^2^ = 0.204; *P* = 0.00001). Similar to the NP microbiota, the greatest dissimilarity among treatment groups occurred at days 2 and 5 (Fig. 3b). Subsequently, the cohorts were more similar to each other, although the oxytetracycline group at day 34 was more dissimilar to the control animals than to the tulathromycin-treated cohort. As with the NP microbiota, overall, the structure of the fecal microbial community was more affected by time (*R*^2^ = 0.091; *P* = 0.00001) than antibiotic treatment (*R*^2^ = 0.029; *P* = 0.00001) from day 2 through 34.

In comparison with the control group, treatment with oxytetracycline significantly decreased the abundance of 27 OTUs at 2 days post-treatment, including those classified as *Alloprevotella*, *Bacteroides*, *Rikenellaceae* RC9 gut group, and *Sutterella* (FDR < 0.05; Additional file 1: Table S5). *Sutterella* and *Rikenellaceae* RC9 gut group were also among the five OTUs reduced in the fecal microbiota of oxytetracycline cattle at day 5. Only two OTUs were enriched in the oxytetracycline cattle at days 2 and 5. By day 12, only five OTUs were differentially abundant between the control and oxytetracycline-treated animals and on the last sampling day (day 34), only a single OTU was depleted in the oxytetracycline group, suggesting at least a partial recovery of the fecal microbiota. Similar to the oxytetracycline-injected cattle, there were 20 OTUs that were significantly reduced in abundance in the tulathromycin group compared with the control cohort at day 2 (FDR < 0.05; Additional file 1: Table S6). An OTU classified as a member of the *Porphyromonadaceae* family strongly depleted in the tulathromycin-treated cattle at days 12 and 19. There were also six OTUs that were differentially abundant at day 34 but none had a relative abundance greater than 0.25%.

In contrast to the NP microbiota, the OTU richness and inverse Simpson’s diversity decreased significantly following feedlot placement (*P* < 0.05; Additional file 4: Figure S3). The fecal microbiota of the oxytetracycline cattle had a significantly lower OTU richness than the control group, but only at day 2 (*P* < 0.05). Overall, *Bacteroides*, the *Rikenellaceae* RC9 gut group, and members of the *Ruminococcaceae* family were the most relatively abundant genera (Additional file 5: Figure S4). Most notably, *Succinivibrio* increased from less than 0.1% at both d minus 5 and day 0 to greater than 4.5% relative abundance on day 2 through day 34. The relative abundance of *Prevotella* also increased from less than 0.01% at d minus 5 to greater than 1% at day 0 onward.

### Antibiotic resistance determinants

**Fig. 6:**
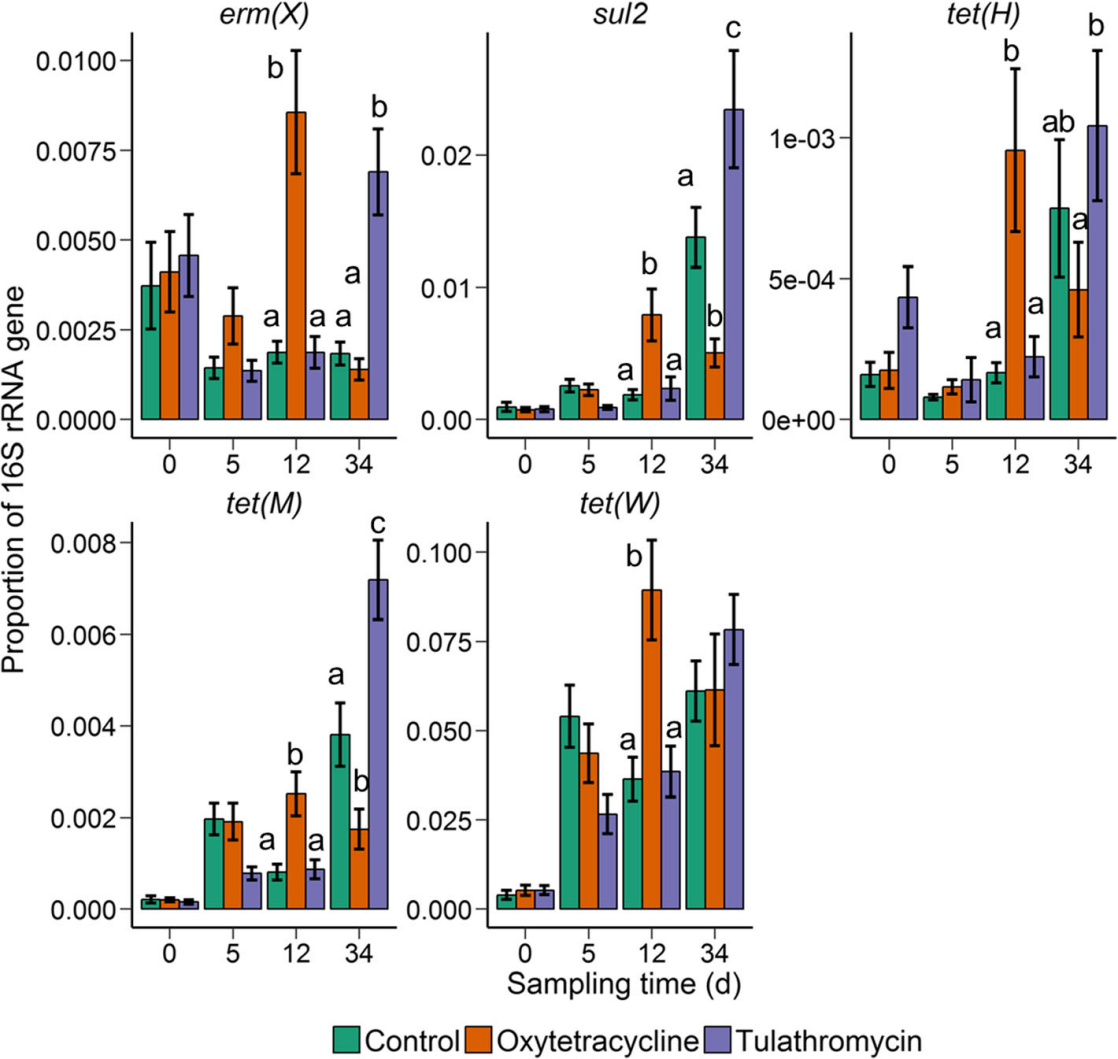
The proportion of the resistance determinants *erm*(X), *sul2*, *tet*(H), *tet*(M), and *tet*(W) to 16S rRNA gene copies in nasopharyngeal samples within each treatment group at days 0, 5, 12, and 34. Different lowercase letters within each sampling time represent significantly different means (*P* < 0.05). Error bars indicate ± standard error of the mean (*n* = 12)

We attempted to quantify the proportions of ten antibiotic resistance determinants in fecal and NP samples from four sampling times; days 0, 5, 12, and 34. Of these ten resistance genes, only six [*erm*(X), *sul2*, *tet*(C), *tet*(H), *tet*(M), *tet*(W)] were above the limit of detection in either the fecal or NP samples. Oxytetracycline significantly increased the proportion of *erm*(X), *sul2*, *tet*(H), *tet*(M), and *tet*(W), in the nasopharynx at day 12 compared with both the control and tulathromycin-treated cattle (Fig. 6). Interestingly, the tulathromycin group had higher levels of *erm*(X), *sul2*, and *tet*(M), at day 34 when compared with the oxytetracycline and control animals.

**Fig. 7:**
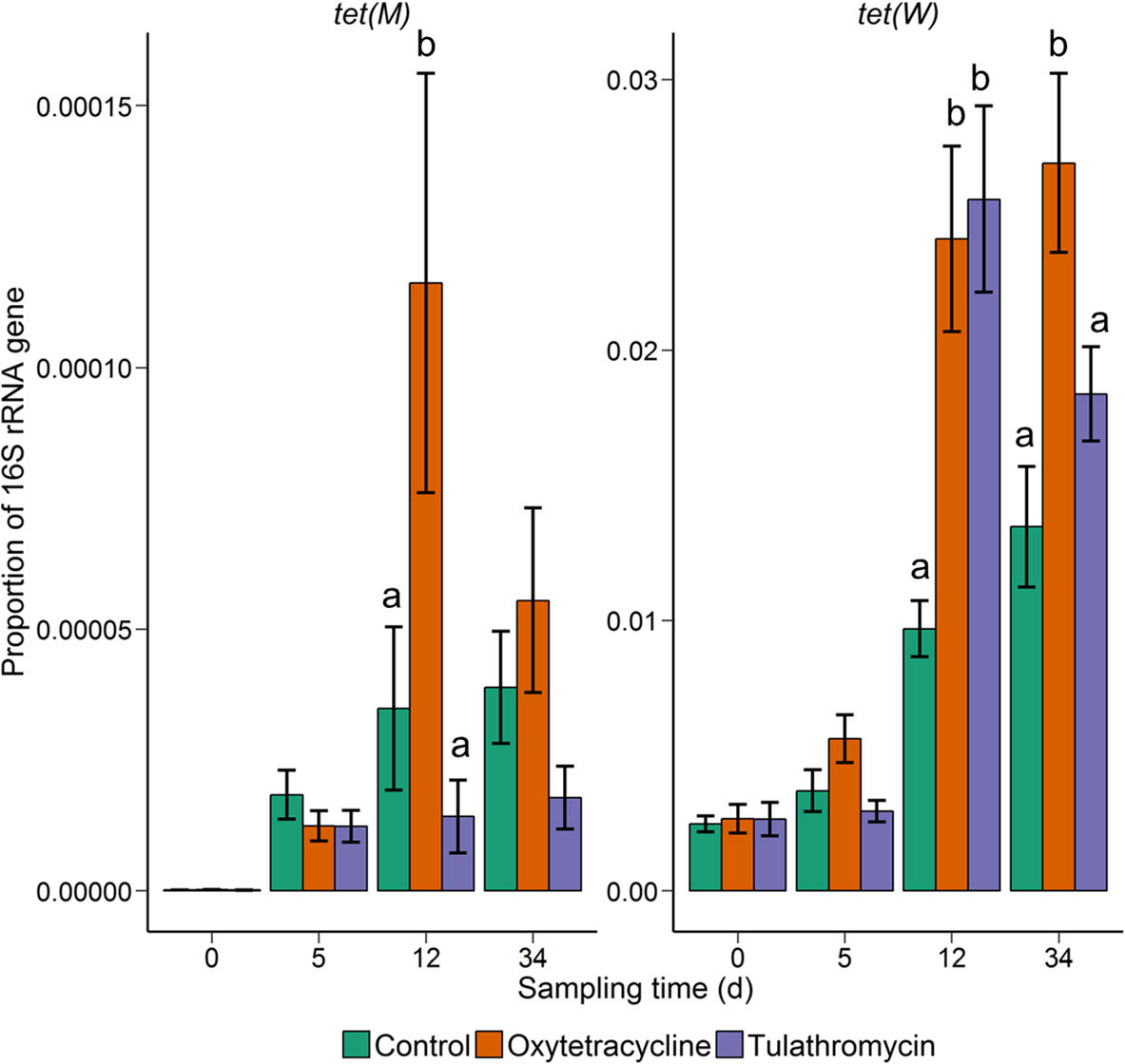
The proportion of the resistance determinants *tet*(M) and *tet*(W) to 16S rRNA gene copies in fecal samples within each treatment group at days 0, 5, 12, and 34. Different lowercase letters within each sampling time represent significantly different means (*P* < 0.05). Error bars indicate ± standard error of the mean (*n* = 12)

Although five resistance genes were detected [*erm*(X), *sul2*, *tet*(C), *tet*(M), and *tet*(W)] in the fecal microbiome, only *tet*(M) and *tet*(W) differed significantly by antibiotic treatment group (Fig. 7). The proportion of *erm*(X), *sul2*, and *tet*(C) to copies of the 16S rRNA gene was below 1.0 × 10^−6^ (data not shown). In the cattle that received an oxytetracycline injection, the relative abundance of *tet*(M) was elevated at day 12 and *tet*(W) at days 12 and 34, in comparison with the control cohort. Tulathromycin also increased the level of *tet*(W) at day 12 compared with the fecal samples from the control animals. Many of the resistance genes were also enriched in NP and fecal samples at day 34 relative to day 0. Among all treatments, the relative abundance at day 34 was significantly higher for *sul2*, *tet*(H) *tet*(M), and *tet*(W) in the NP samples and *tet*(M) and *tet*(W) in the fecal samples compared with the baseline proportions (day 0) (*P* < 0.05).

## Discussion

Feedlot cattle frequently receive an injection of at least one antibiotic during initial processing to control BRD-associated bacteria [2] and therefore it is important to understand the potential unintended consequences on the bovine microbiome and antibiotic resistance. In the present study, we determined the effect that a single injection of either oxytetracycline or tulathromycin has on the fecal and NP microbiota of feedlot cattle. As hypothesized, antibiotic treatment had a larger effect on the NP microbial community structure compared with the fecal microbiota, although this was only true for oxytetracycline-treated cattle. Oxytetracycline and tulathromycin also increased the proportion of several antibiotic resistance determinants in the fecal and NP microbiome (Figs. 6 and 7).

For both oxytetracycline and tulathromycin, the greatest effect on the NP microbiota was observed on days 2 and 5 based on the Bray-Curtis dissimilarities (Fig. 3a). This was not unexpected given that these are the most immediate sampling times following administration and likely when bovine antibiotic concentrations were highest. The lung concentration of oxytetracycline has been reported to peak at 1.3 μg/g at 12 h [29] and tulathromycin at 4.1 μg/g at 24 h [30]. Though data on the active concentrations of these antibiotics in the upper respiratory tract of treated cattle are limited, oxytetracycline has been shown to achieve therapeutic concentrations in oral fluid [31] and nasal secretions [32] of pigs after administration. Given the changes in the NP microbiota that we observed, it is probable that oxytetracycline and tulathromycin penetrated into the intranasal secretions of cattle and achieved concentrations that inhibited certain bacteria.

Both antibiotics used in our study are intended to be long-acting drugs and the NP microbiota remained altered in the oxytetracycline group in comparison with the control and tulathromycin-treated cattle. Oxytetracycline and tulathromycin each appeared to offer some protection against *Pasteurella* spp. colonization in the nasopharynx at days 2 and 5, and oxytetracycline against *Mycoplasma* spp. at day 2 (Additional file 1: Table S2, S3). Treatment of BRD associated with members of these two genera is among the indications for both antibiotics. When used for metaphylaxis to mitigate BRD, antibiotics are thought to reduce pulmonary bacterial load in cattle [33]. Our study showed that the efficacy of metaphylactic antibiotics may also be partially due to reducing the abundance of BRD-associated bacteria in the upper respiratory tract, which is the reservoir of these opportunistic pathogens. A reduction of these bacteria in the nasopharynx would potentially limit proliferation and subsequent inhalation into the lungs. In support of this, studies have shown decreased prevalence of *M. haemolytica* isolated from nasal swabs of cattle administered tulathromycin [34] and tilmicosin [35].

Interestingly, an abundant *Mycoplasma* OTU (9.2%) was enriched in the NP microbiota of oxytetracycline-administered cattle at day 34, indicating that protection against *Mycoplasma* spp. may be only temporary and oxytetracycline use may actually promote a greater abundance of *Mycoplasma* later in the feeding period. A randomized study by Hendrick and colleagues [36] found that although calves receiving metaphylactic oxytetracycline at feedlot arrival had a reduced risk of BRD, they also had an increased risk of arthritis. Chronic pneumonia and polyarthritis syndrome (CPPS) is a disease associated with *M*. *bovis* and typically occurs later in the feeding period. Although we only identified *Mycoplasma* at the genus level, the association we observed between oxytetracycline and *Mycoplasma*, in conjunction with the study by Hendrick et al. [36], indicates that additional studies are warranted to further evaluate a potential causal link between oxytetracycline and the incidence of CPPS.

An OTU in the NP microbiota that was classified at the family level as *Microbacteriaceae* was also strongly affected by antibiotic treatment. It was reduced in both treatment groups compared to the control animals at day 12, and also at days 2 and 5 in the oxytetracycline cohort. This OTU is particularly notable because its relative abundance was greater than 3.6% at days 2, 5, and 12 in the control cattle but less than 0.02% and 0.80% in the oxytetracycline and tulathromycin-treated animals, respectively. Furthermore, the relative abundance of this OTU was greater than 2.3% at days 0 and 0.95% at days 19 in all groups (data not shown). This finding suggests that this *Microbacteriaceae* member is especially sensitive to both antibiotics and that it is also able to re-establish itself within the nasopharynx as antibiotic concentrations decrease. The genera in this family are Gram-positive aerobes and are typically associated with the soil environment [37]; however, previous work has identified several genera within this family in bovine NP samples in high abundance [8, 11, 38–40]. In an earlier study, we also isolated a member of this family, *Microbacterium*, from the nasopharynx of untreated feedlot cattle [17]. Similarly, an OTU within the Sphingobacteriales order (phylum Bacteroidetes) was significantly depleted in the oxytetracycline NP microbiota compared to the control cattle.

Overall, the fecal microbiota was less affected by antibiotic treatment than the NP microbiota (Fig. 2). Although the fecal microbiota of the three treatment groups was most dissimilar to each other at days 2 and 5, beyond this sampling time they were actually more similar than they were at day 0. In addition, only four OTUs were differentially abundant in the fecal microbiota of the control and oxytetracycline cattle at either day 19 or 34 (Additional file 1: Table S5) and only one OTU having a relative abundance greater than 0.3% was differentially abundant in the tulathromycin and control cattle at these last two sampling times (Additional file 1: Table S5). However, at day 2, both the oxytetracycline and tulathromycin treatment groups had a significantly lower abundance of OTUs identified as *Alistipes*, *Alloprevotella*, *Bacteroides*, Prevotellaceae UCG-001, *Sutterella*, and the RC9 gut group in their feces compared with the control animals. In contrast, *Phocaeicola* was significantly enriched in the fecal microbiota of the tulathromycin cattle at day 5 and is a relatively new genus with currently only a single species; *Phocaeicola abscessus* [41]. This genus has been reported to be abundant in both bovine rumen [42] and fecal samples [43]. *Alistipes* and the RC9 gut group are both members of the *Rikenellaceae* family, which is also in the same order (Bacteroidales) as *Alloprevotella*, *Bacteroides*, and *Prevotellaceae* UCG-001. Bacteria in the Bacteroidales order are strongly associated with the mammalian gastrointestinal tract [44]. Interestingly, *Alistipes*, *Bacteroides*, and *Sutterella* OTUs have also been reported to be reduced in the fecal microbiota of humans treated with ciprofloxacin [45]. *Alloprevotella* [46] and the *Rikenellaceae* family [47] are both relatively new taxonomic groups, and some members of the *Rikenellaceae* are producers of the short-chain fatty acids acetate and propionate, the latter being the most important energy source for cattle.

By far the largest change in the fecal and NP microbial communities was observed between d minus 5 and day 0 (Figs. 1 and 3). During this period, the cattle were transported from the research farm to the feedlot, a distance of 20 km. This finding is in agreement with our earlier study where the NP microbiota of cattle from this same herd also shifted significantly 2 days after transport and continued to change until at least 7 days post-arrival [8]. Stress associated with transport, handling, and introduction to a new environment is likely a factor in these changes. The risk for developing BRD is highest at feedlot placement and elevated levels of serum cortisol and neutrophils have been reported in cattle immediately following transport [48, 49]. A recent study by Deng et al. [50] found that the concentrations of certain bacterial species were altered in the rumen of transported cattle with a concomitant increase in circulating adrenocorticotropic hormone and cortisol. In addition, differences in the ADF content of the barley silages at the farm and feedlot may also have contributed to the large alterations in the fecal microbiota that we observed.

In accordance with our previous study [8], the OTU richness and microbial diversity of the NP microbiota increased significantly when the cattle were transferred to the feedlot (Additional file 2: Figure S1). Among the OTUs that were found to be enriched at day 0 were those classified as *Acinetobacter*, *Clostridium*, *Corynebacterium*, *Psychrobacter*, and *Streptococcus* (Additional file 1: Table S1). These genera are often abundant in the NP microbiota of feedlot cattle [8, 40, 51]. Certain species within *Acinetobacter* [52], *Clostridium*, and *Streptococcus* [53] have the ability to degrade and utilize mucin. It is interesting to speculate whether their increase at feedlot arrival indicates a disturbance in the upper respiratory mucosa. This could have relevance to pathogen growth and BRD development, and should be investigated further as limited information is available on how mucosal health relates to the microbiota in cattle. Although *Moraxella*, *Mycoplasma*, and *Pasteurella* are also commonly prevalent in feedlot cattle [8, 40, 51], they were reduced in abundance from d minus 5 to day 0 when cattle arrived at the feedlot. OTUs within the Sphingobacteriales order were also associated with the d minus 5 samples. One of these Sphingobacteriales OTUs was classified as belonging to *Filobacterium*, a new genus that has been linked to respiratory disease in rodents [54]. However, we have also recently detected this genus in high abundance in the nasopharynx of individual commercial feedlot cattle [11] and so the biological significance of this order in the bovine respiratory tract remains unclear.

In contrast to the microbial diversity of the nasopharynx, the OTU richness and inverse Simpson’s diversity decreased in the fecal microbiota after transport and remained reduced throughout the study period (Additional file 5: Figure S4). A reduction in OTUs classified as members of the *Alistipes* and *Phocaeicola* genera and the *Ruminococcaceae* family was associated with this shift from d minus 5 to day 0, as was an increase in *Clostridium*, *Escherichia/Shigella*, *Prevotella*, and *Rikenellaceae* RC9 gut group OTUs (Additional file 1: Table S4). It is difficult to ascertain what caused these alterations, especially since cattle were fed barley silage both at the farm and feedlot. However, despite DM, OM, and NDF being similar between the silages, the silage at the feedlot had lower ADF content, indicating greater digestibility. Similar to our study, the abundance of the family *Ruminococcaceae* was lower and the genera *Prevotella* and *Clostridium* higher in the feces of cattle fed diets with greater digestibility [55]. Thus, the lower digestive tract microbiota of cattle is responsive to dietary changes. Although it is not known why the NP and fecal microbiota responded differently to feedlot placement, it may be due to anatomical reasons. That is, the nasopharynx is much more likely to be exposed to novel microorganisms through aspiration and contact with other cattle and the soil than the lower gastrointestinal tract. The microbial communities of each anatomical region may also respond differently to physiological stress that calves would experience over the course of transportation and introduction to a new feedlot.

We also attempted to quantify ten different antibiotic resistance determinants in the fecal and NP samples at days 0, 5, 12, and 34 (Figs. 6 and 7). Of the six resistance determinants detected, the proportion of *tet*(M) and *tet*(W) was affected by antibiotic treatment in both fecal and NP samples. These two genes were also more relatively abundant in all groups at day 34 compared to day 0. *tet*(M) and *tet*(W) are abundant in fecal [56] and manure [57] samples from feedlot cattle and also in the airborne particulate matter of the feedlot environment [58], demonstrating the potential for transmission in feedlots. Both oxytetracycline and tulathromycin increased the proportion of resistance determinants in the NP microbiome, albeit at different time points. This included the sulfonamide resistance gene, *sul2*, which is not associated with macrolide or tetracycline resistance. However, the administration of one antibiotic can provide selective pressure for the maintenance of other unrelated resistance determinants through linkage on mobile genetic elements. For example, the cotransfer of *erm*(B) and *tet*(M) in the presence of the macrolide erythromycin has been described in *Streptococcus pyogenes* isolates [59]. Additionally, many resistance genes have been reported to be colocalized on mobile genetic elements found in swine fecal and manure samples [60].

The relative abundance of the *sul2*, *tet*(H), and *tet*(M) in the NP microbiome was lower than our recent study using commercial feedlot cattle [11], perhaps because the cattle in the present study were sourced from a closed herd with no history of in-feed antibiotic use. Therefore, it was expected that these cattle would have lower concentrations of antibiotic resistance determinants than commercially sourced cattle which can be exposed to antibiotics at various times during production and/or may also acquire microbiota from animals that had been given antibiotics. A 2013 study by Zaheer et al. [34] had demonstrated that a single injection of tulathromycin (2.5 mg/kg body weight) significantly increased the percentage of erythromycin-resistant enterococci in the feces of feedlot cattle 7 days post-treatment. In Alberta, antibiotic resistance among BRD pathogens is increasing [61] and high percentages of resistance (> 70%) to oxytetracycline and tulathromycin have recently been reported for *M. haemolytica* and *P*. *multocida* isolated from feedlot cattle [62]. Consequently, although the risk of dissemination into the environment is greater through fecal shedding, resistant bacteria and resistance genes in the NP microbiome should also be a concern since they may be transmitted among cattle and potentially increase the incidence of antibiotic treatment failure.

## Conclusions

This study demonstrated that the fecal and NP microbiota of beef cattle is significantly altered 2 and 5 days after a single injection of either oxytetracycline or tulathromycin at feedlot placement. For the antibiotics studied, the fecal microbiota appears to be more resilient to antibiotic treatment than the NP microbiota, although the NP microbiota of the tulathromycin-treated cattle also recovered by day 12. Although both antibiotics reduced known BRD-associated pathogens, an effect on the abundance of other bacteria was also evident. The consequences of these collateral effects are not known. The initial decrease in *Mycoplasma* spp. after oxytetracycline administration, followed by an increase later in the feeding period, may be an undesirable effect should this have implications for CPPS. A large shift in the fecal and NP microbiota was also observed following transport to the feedlot. More research on changes in the bovine respiratory mucosa during transportation may provide important information on factors that alter the microbiota. Oxytetracycline and tulathromycin also increased the proportion of several antibiotic resistance genes in the fecal and NP microbiome relative to the control animals, albeit at different times.

## Additional files

Additional file 1: **Table S1.** Differentially abundant OTUs in the nasopharyngeal microbiota following transport to the feedlot (d minus 5 vs. d 0). **Table S2.** Differentially abundant OTUs in the fecal and nasopharyngeal microbiota between antibiotic-treated and control cattle at d 5. **Table S3.** Differentially abundant OTUs in the fecal microbiota following transport to the feedlot (d minus 5 vs. d 0). **Table S4.** Differentially abundant OTUs in the fecal microbiota following transport to the feedlot (d minus 5 vs. d 0). **Table S5.** Differentially abundant OTUs in the fecal microbiota between the oxytetracycline-treated and control cattle. **Table S6.** Differentially abundant OTUs in the fecal microbiota between the tulathromycin-treated and control cattle. (XLSX 52 kb)

Additional file 2: **Figure S1.** Box and whisker plots of the number of OTUs (A) and inverse Simpson’s diversity index (B) in the nasopharyngeal microbiota by sampling time and treatment group. Different lowercase letters within each sampling time represent significantly different means (*P* < 0.05). Error bars indicate ± standard error of the mean (*n* = 12). The box in the box plots indicates the interquartile range (IQR) (middle 50% of the data), the middle line represents the median value, and the whiskers represents 1.5 times the IQR. (TIFF 936 kb)

Additional file 3: **Figure S2.** Box plots displaying the percent relative abundance for the ten most relatively abundant genera in the nasopharyngeal microbiota by sampling time and treatment group (*n* = 12). (TIFF 963 kb)

Additional file 4: **Figure S3.** The number of OTUs (A) and inverse Simpson’s diversity index (B) in the fecal microbiota by sampling time and treatment group. Different lowercase letters within each sampling time represent significantly different means (*P* < 0.05). Error bars indicate ± standard error of the mean (*n* = 12). (TIFF 745 kb)

Additional file 5: **Figure S4.** Box plots displaying the percent relative abundance for the ten most relatively abundant genera in the fecal microbiota by sampling time and treatment group (*n* = 12). (TIFF 1076 kb)

## Abbreviations

ADF: Acid detergent fiber analysis
BRD: Bovine respiratory disease
CPPS: Chronic pneumonia and polyarthritis syndrome
DM: Dry matter
NDF: Neutral detergent fiber
NP: Nasopharyngeal
OM: Organic matter
OTU: Operational taxonomic unit
