# Supplementary figures and images for: Antibiotic treatment in feedlot cattle: a longitudinal study of the effect of oxytetracycline and tulathromycin on the fecal and nasopharyngeal microbiota
Source: Microbiome. 2019 Jun 5;7:86. doi: 10.1186/s40168-019-0696-4 (PMC6549328; doi:10.1186/s40168-019-0696-4)

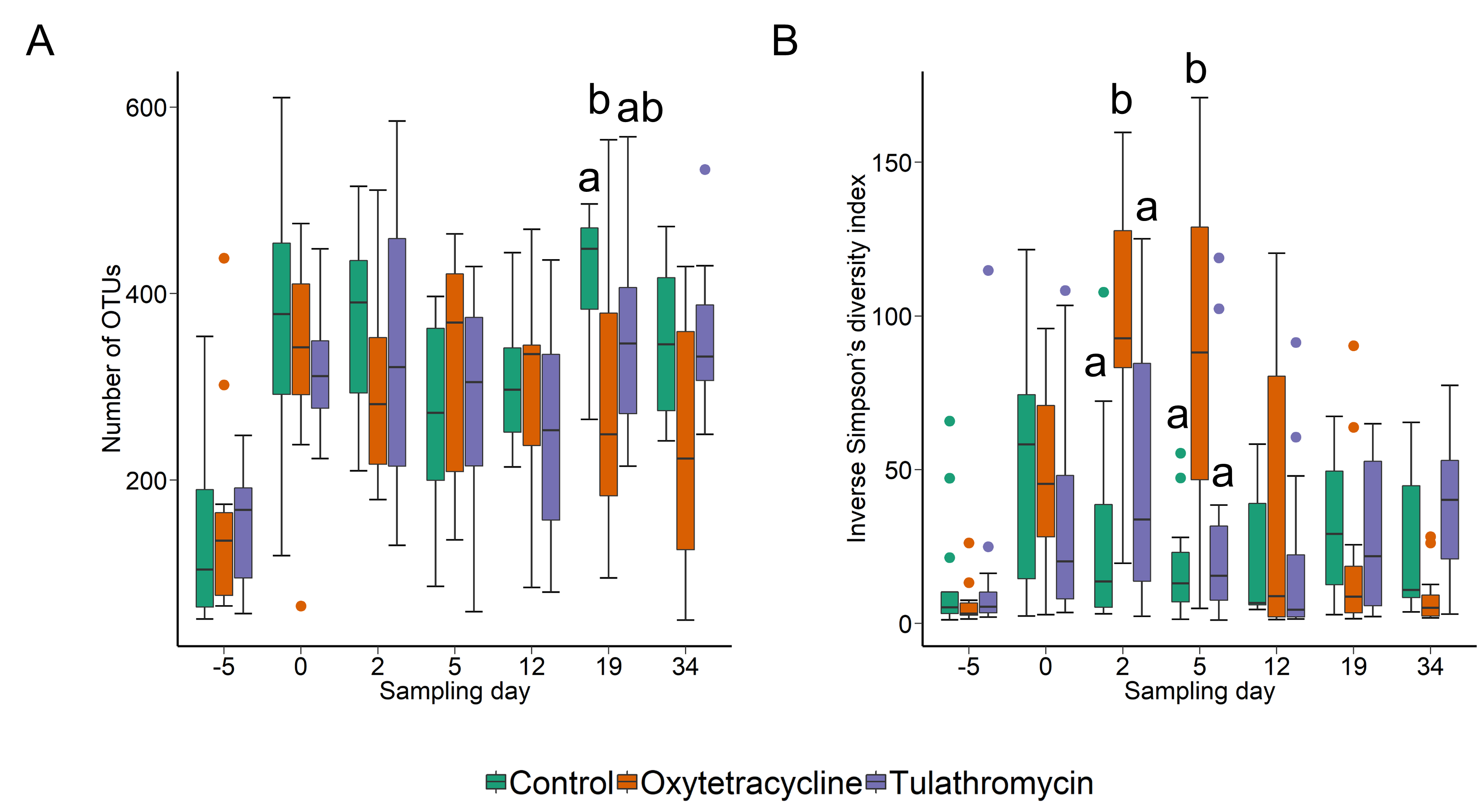

Supplement: Supplementary file 2 — Figure S1. Box and whisker plots of the number of OTUs (A) and inverse Simpson’s diversity index (B) in the nasopharyngeal microbiota by sampling time and treatment group. Different lowercase letters within each sampling time represent significantly different means (P < 0.05). Error bars indicate ± standard error of the mean (n = 12). The box in the box plots indicates the interquartile range (IQR) (middle 50% of the data), the middle line represents the median value, and the whiskers represents 1.5 times the IQR. (TIFF 936 kb) [file 40168_2019_696_MOESM2_ESM.tiff]

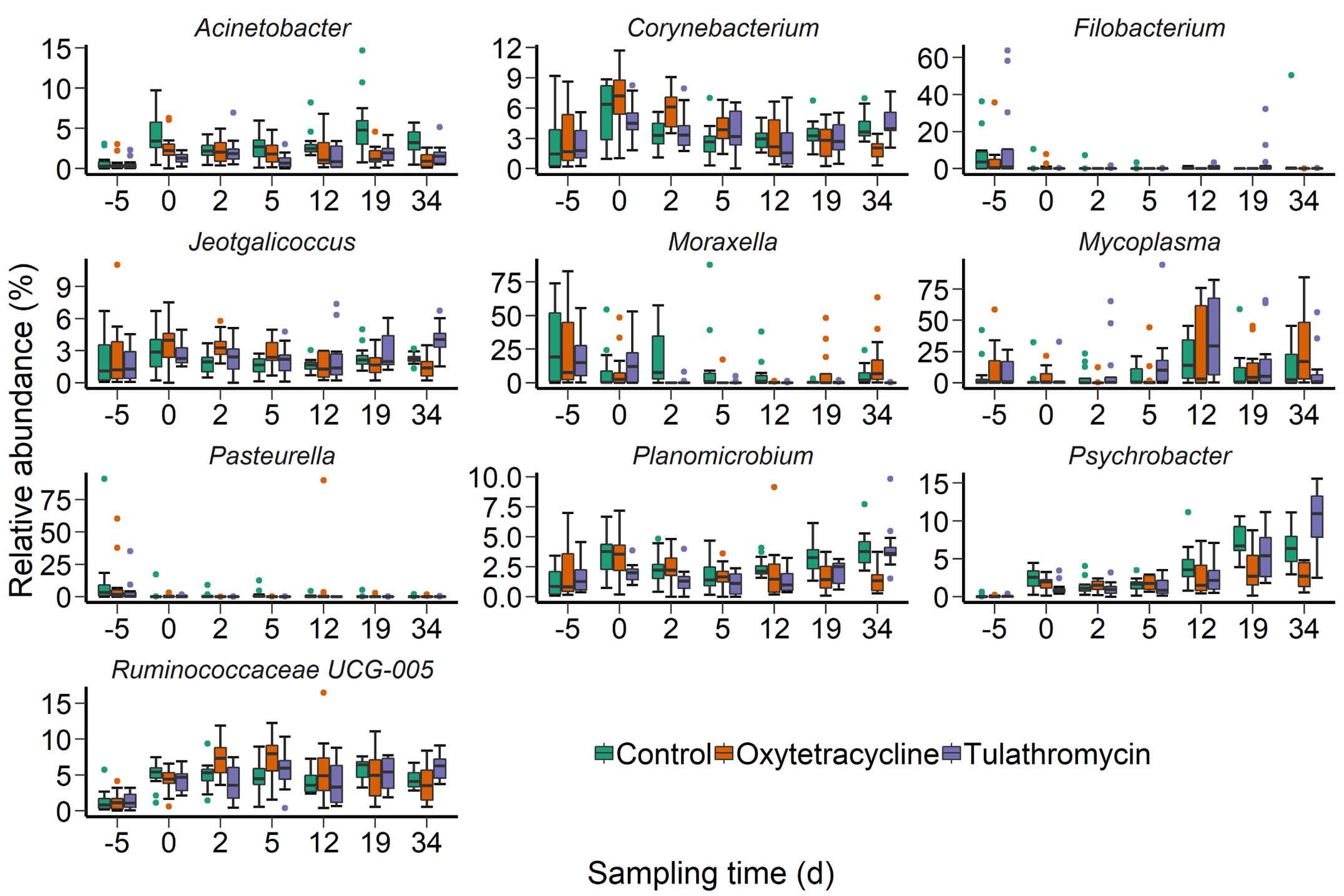

Supplement: Supplementary file 3 — Figure S2. Box plots displaying the percent relative abundance for the ten most relatively abundant genera in the nasopharyngeal microbiota by sampling time and treatment group (n = 12). (TIFF 963 kb) [file 40168_2019_696_MOESM3_ESM.tiff]

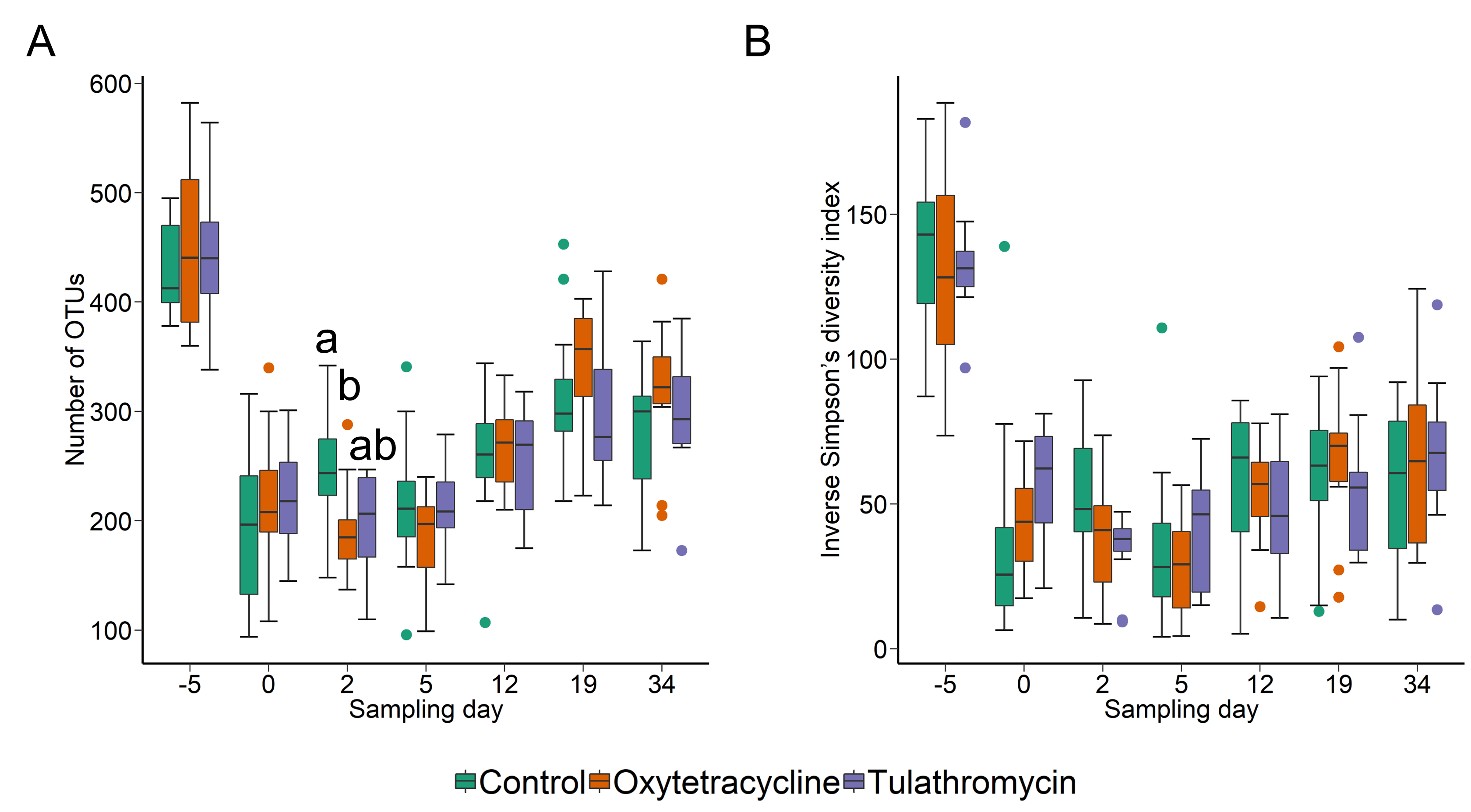

Supplement: Supplementary file 4 — Figure S3. The number of OTUs (A) and inverse Simpson’s diversity index (B) in the fecal microbiota by sampling time and treatment group. Different lowercase letters within each sampling time represent significantly different means (P < 0.05). Error bars indicate ± standard error of the mean (n = 12). (TIFF 745 kb) [file 40168_2019_696_MOESM4_ESM.tiff]

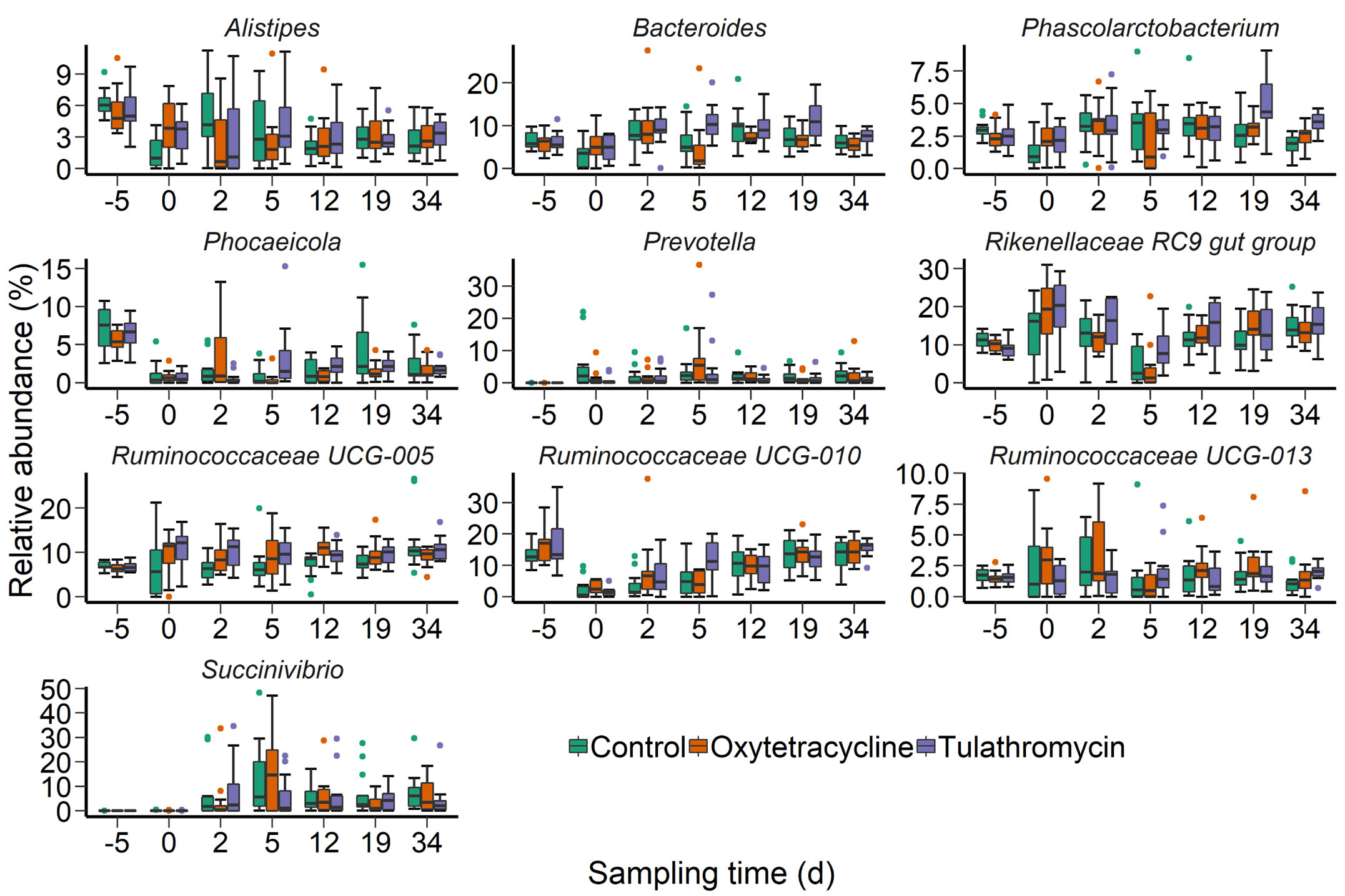

Supplement: Supplementary file 5 — Figure S4. Box plots displaying the percent relative abundance for the ten most relatively abundant genera in the fecal microbiota by sampling time and treatment group (n = 12). (TIFF 1076 kb) [file 40168_2019_696_MOESM5_ESM.tiff]
